# Case of Convulsions Depending on Various Eccentric Causes

**Published:** 1852-12

**Authors:** S. B. Hunt

**Affiliations:** Mendon, Monroe Co., N. Y.


ART. II. — *Case of Convulsions depending on various Eccentric Causes.* By S. B. Hunt, M. D., Mendon, Monroe Co., N. Y.

Not from diseases of common and acknowledged type, but from *exceptional* cases, derive we the strongest and most faithful light.

It is not my purpose in placing this case upon record, to add another to the useless histories of fancied anomalies in disease which crowd the pages of our journals — although in duration and intensity of pain, in violence and peculiarity of spasm, it has rarely been equaled — although it had many phenomena of almost inscrutable obscurity — yet these facts alone are worthless compared with the greater fact, that in it are illustrated and made plain many of the deeper mysteries of neuralgic disease.

The reader will pardon me for minuteness of detail, not only of symptoms, but of the various peculiarities of mental and physical organization which characterize this patient; for to those idiosyncrasies must we look for the rationale of symptoms, and the *origo mali* of disease. So strong is my conviction of the influence of mental causes in, (not producing) but in *determining the character* of disease, that I wish if possible to bring to light those influences, and the manner in which they operate.

William Sherry, the subject of this report, is a farm laborer, age twenty-one years. His temperament is nervo-bilious. Possessed of a saturnine disposition — a man of few words — slow and dull in scholarship — indifferent in his attachments — he performed his duties as a laborer without hurry or enthusiasm, but faithfully, and mechanically. His brain is neither large nor active, and his eccentricities of character being such as to attract much notice, he has been in some measure the butt of his schoolmates, and the subject of their practical jokes.

With such a cerebral organization, he has the usual accompaniment of a fully developed spinal system. His frame is heavy built and muscular; height five feet eleven inches; weight about 170, when in full health. Though rather slow in motion, he is possessed of more than the average strength. It will be seen from this description that his spinal system is not so fully under the control of the brain as it should be when there is a proper balance of power between those two great nervous centers.

He applied to me in June last. His symptoms indicated hepatic obstruction, and on examination his liver was found considerably enlarged. The diagnosis was fatty deposit in the liver from a deficiency of the alkaline carbonates in the blood. Bicarbonate of soda was the leading prescription, and under this his side diminished in size, and his general symptoms improved correspondingly. Coming one day to my office he complained of strange feelings — lost for a moment his consciousness — breathed heavily — then slight cramps with violent trembling followed, and in a moment he was himself again. There was no change in the pulse, or paleness of surface. Having seen this form of convulsion in other cases of hypertrophied liver, I did not make it a subject of treatment. These slight convulsions occurred regularly, sometimes in church. During them his lips were blue, face flushed, and swollen. Somewhat epileptic in character, I considered them as the the *petit mal* of the French.

While convalescent from his liver disease, an herpetic eruption appeared upon the testicles with a most intolerable sense of burning and itching, in bearing which he manifested a lack of fortitude, raving about the room during the paroxysms in a perfect frenzy of excitement. At one time I dressed the eruption, applying a cooling saturnine lotion. Notwithstanding its mild and soothing character, he manifested the keenest suffering from its application. Leaving the room for a moment, on returning I found him on the floor frothing at the mouth, but conscious and able to help himself on to the bed. The same evening I was called to him and found him in a convulsion. His countenance was tinged and swollen. He came out of it in great pain, and seeing a tendency to a return of the fit I bled him 16 oz. This relieved him, and I placed him upon the valerianate of zinc with blue mass.

This eruption receded in a day or two, and was followed by retention of urine which yielded to my favorite diuretic, the iodide of potassium. Soon after, the eruption came out again, then receded, and he came to my office hardly able to walk from distention of the bladder. The introduction of the catheter caused considerable pain, and soon after the urine was drawn off, he complained of "strange feelings" again, and went off in one of the most violent epileptic convulsions it was ever my lot to witness. I prescribed again iodide of potassium for the inflammatory action in the mucous coat of the bladder, with blue mass and zinc valerian as a general alterative and nervous tonic. This was early in August. After a few days' use of these drugs, he apparently recovered and wished to return to his work but yielded to my persuasions to keep quiet.

*Aug.* 30*th*, *evening.* I found him in a most violent convulsion, which was rapidly repeated. Coming out of it he complained of cramp and pain in the stomach. On inquiry I found that he had eaten immoderately of green fruit during the day. An emetic dislodged a quantity of apples, green corn, cucumbers and beets, in large undigested, and unmasticated pieces. The relief was only partial. Calomel, grs. xx, jalap, grs. xv, was then given.

Later in the evening his symptoms were coldness of the extremities, extreme pain in the epigastrium, and cramp in the stomach and bowels, which organs could be felt beneath the abdominal muscles as rigid as iron bars. This cramp extended by sympathy to the voluntary muscles, until losing consciousness he was plunged into the most uncontrollable fits. Opiates and anti-spasmodics were administered *pro re nata.*

The case had now become pronounced. The small aberrations from muscular control which we have called *petit mal*, have magnified themselves into the *grand mal.* He has epilepsy from eccentric causes, and in a form of unusual severity. He knows no relief from pain so severe as to cause the most terrific outcry, save when he loses sensation in a convulsion equally terrible. It is no unusual thing for crude food to cause cramp, but why should this cramp extend to the voluntary muscles, until gathering strength, it sweeps down all the barriers of mental control and consciousness? The answer is to be found in the fact of his strong spinal system and weak brain. An intellectual man of more feeble frame, would bear this pain without yielding up to any great extent his control over the voluntary muscles. This statement will find confirmation in the further history of the case.

*Aug.* 31*st.* Vomited during the day quantities of undigested fruit with some relief. Movements of the bowels occurred and he was more easy though having occasional returns of convulsion till

*Sept.* 1*st.* All the symptoms returned with increased violence. There was, in the character of the fits some variations from true epilepsy. The *aura* was distinct in the shape of pain in the bowels, and sometimes prolonged for many minutes. Sometimes during the *aura*, I sat by him exhorting him to fortitude under his sufferings, and to efforts of the will to keep off the fits. Sometimes by bringing all his resolution to bear, he was able to stave off the paroxysm, but as the pain increased in intensity, he gave way, and found relief in the lethe of unconsciousness. For four or five days, at least one half of his time was spent in convulsion, the other half in pain. Generally after the fit there was heavy inspiration, and then delirium, during which he recollected the events of his fit, but saw them in a distorted point of view, manifesting the greatest antipathy to those who had held him on the bed, and much affright when those persons came into the room — the fright being sometimes sufficient to cause renewed convulsion. Afterward there was during the paroxysm a kind of semi-consciousness, like that observed in hysterics—and this reminds me that his urine (which was removed entirely by the catheter) was very copious and limpid.

The treatment had two indications. First, to control pain and so prevent convulsion; and secondly, to get rid by calomel and cathartics of that inflammatory condition of the mucous membranes of the bowels and bladder which kept up the pain. Now this inflammation was the ordinary follicular enteritis of a mild grade. It is difficult to imagine how so slight an inflammation could cause so severe pain; unless it was by causing neuralgia of the internal viscera, and we find the counterpart to this in the intense pain and convulsions accompanying some trivial uterine affections. From the night of Sept. 1st up to Monday, Sept. 6th, morphine was given in *one grain doses, hourly*, decreasing, however, to half grain for the last day or two. Calomel was given in alterative doses, and free action of the bowels secured by the cold saline enema. Nothing afforded so much relief as these last. They brought away quantities of tough gum water phlegm, invariably followed by temporary relief of the symptoms. The stools gradually became more fœcal and consistent as he improved. Of course all the usual adjuvants of mustard, friction, baths, and anti-spasmodics were put in force. On the 6th the Sulph. Morph. was suspended and 60 drops Tinct. Opii given hourly. Sept. 7th, 45 drops hourly. Sept. 8th, ordered to be given pro re nata with a decrease in quantity. Only ¼ oz. was taken during the ten days.

*Friday Sept 10th.* The symptoms now remaining are those of follicular enteritis.

℞ Calomel, gr. j., night and morning.
Salacine, gr. v., before meals.

*Sunday, Sept. 12th.* Patient is up and about the house, appetite good, tongue clean, but still some tenderness about the bowels. Continue treatment.

After a few days my patient resumed labor, and continued in good health till October 15th, when he was taken with a fit in a store. It was violent, and he had two others on his way home. I could learn of no imprudence of diet, but the symptoms (less the nausea) were those of the previous attack.

Calomel, gr. xx, with Jalap, gr. xv, was given at 7 o'clock, P. M., and repeated at 12. A pill of calomel and conium was given every hour. Towards morning he grew easier, and having free movements of the bowels he escaped with only one or two convulsions until evening, when they came on again. Accompanying, simultaneous, increasing, and decreasing with the pain in the bowels, was intense pain in the occipital region. There was tenderness of three or four of the cervical vertebræ. A large blister was applied to the nuchæ. At midnight, after six hours of continued convulsion and pain, he took 8 grs. of quinine, grew easy in about fifteen minutes, and though still suffering with the pain in the head, was tolerably easy.

I have neglected to mention, that during the evening of the 15th I took something more than two pints of blood from the arm, without materially affecting the force of the circulation, or benefiting the patient.

During the 17th, 18th, 19th and 20th, the treatment was made up thus, with occasional increase of the doses during severe pain.

Quinine, gr. viii, every four hours.
Laudanum, gtts. xlv, every two hours.
Calomel, gr. iii, every four hours.

Tinc. Valerian was also given in $\frac{1}{4}$ gr. doses.

The quinine was the most efficient and spasmodic given. The laudanum produced hardly any appreciable effect, even when given in doses much larger than that stated. The calomel acted with its usual certainty upon the inflammatory action, and was evidently an important item in the treatment. Under this management the fits decreased in frequency, and the pain assumed a periodical neuralgic character, coming on at sundown and lasting till 2 o'clock on the night of the 17th, but leaving earlier each night. Free action of the bowels was kept up by enema, and the urine was once or twice a day drawn off with the catheter. It was evident that the pain and spasm would not leave him so long as any inflammatory action continued.

The treatment was accordingly kept up until catharsis was obtained without the use of the syringe. His tongue cleaned off, and he passed the night of the 21st without spasm, and with but slight pain. On the morning of the 22d he reports freedom from pain in the head, and comparative comfort in the bowels. The calomel was suspended — the laudanum ordered given pro re nata — 5 grs. salacine to be given every six hours, alternating with a pill composed of Tinc. Valerian, gr. $\frac{1}{4}$, Pil. Hydrarg. gr. ii.

The patient is now again convalescent. The final issue of the case is not in my mind doubtful. Should he continue a mild alterative, tonic, and nervine treatment, until every vestige of inflammatory action is subdued; and should he conjoin with this several months of rest from labor, with the exposure to wet and cold which farm labor involves, he will regain a state of robust health. But on the other hand, should he be guilty of a renewal of those imprudences which brought on this second attack, he will have a renewal of epileptic seizures, and with his peculiar *physique,* a complete destruction of his mental powers must be anticipated.

I feel that I have already extended this report to a length which may be tedious, but I am unwilling to leave the subject until I have stated those deductions which may be rationally drawn from it. But not on this case alone do these deductions depend. They are the offspring of some study of neuralgic disease in books, some at the bedside, and more than either in the lonely rides which are the lot of the country physician.

1st. There is such a thing as a proper balance of power between different organs having diverse but similar functions, and when one such organ predominates over the other in activity and strength, the result is a variation from true health.

2d. When, there being a want of proper balance and equality between the brain and spinal cord, the brain has the predominance, we shall find an infirm physical development, and the tendency will be towards diseases not convulsive.

3d. When the reverse obtains, (the spine having the predominance) we shall find a tendency to those diseases in which chill, congestion, and convulsion are prominent symptoms.

These statements involve a brief inquiry into the nature of those three conditions which I have linked together in the latter proposition. But prior to, and the precursor of these conditions, is another to which common consent has given the name of irritation.

Now what is irritation? I have heard serious objections to the word on the ground of its indefiniteness. A certain lexicon on my shelves defines it to be "the effect following the application of an irritant." This is very lucid — as clear as Carlyle — but the inquiring mind seeks for something less concise, and more satisfactory. To my mind, that which Kaltenbrunner calls the "period of incubation" — a lapse of time occurring after the application of an irritant, and before the manifestation of congestion — defines the word. Its symptoms are pain, tenderness on pressure, and disordered sensations; the other essentials of inflammation being wanting.

*Ubi irritatio ibi fluxus.* Congestion follows the period of incubation. It is essentially distinct from irritation itself in being a *visible* change in the size and action of the capillary vessels. Closely connected with congestion is that phenomenon called chill. What is chill? Evidently the manifestation of a disturbance of the spinal system, consequent upon the presence of an undue quantity of blood upon the spinal and ganglionic systems, producing that tremor of the muscles, and (by derivation from it) that coldness of the surface, called chill.

This presence of blood in undue quantity is congestion.

If the congestion is intense and long continued as in congestive fever, the chill is permanent, and may never leave the patient until the chill of death is substituted. Or as in cerebro spinal meningitis, it may deepen into that more pronounced and alarming form of chill — convulsion.

These conditions then are but different stages of the same action. Irritation is the prodrome of congestion. Chill is a symptom of congestion, and spasm is but a magnified chill.

Such being the main facts on which we base our argument, let us support them by a cursory examination of the influence of sex, age, physical and mental conformation in the production of disease.

In the child the principal office of the brain is to govern the motive power. It has not yet attained to its higher intellectual function.

A child rarely has chill — in fact those causes which would suffice to cause chill only in the adult, will give us convulsion in the infant. Children are more subject to cerebral, than to spinal disease. The child has not learned to use its muscles — the will has no control over them — there is a lack of that proper balance of power between the brain and spinal cord, which alone can insure a quality of action. In the adult, chill takes the place of convulsion because the brain has acquired such power as enables it by efforts of the will to control those spasmodic actions to which the child must submit.

Women are much more liable to convulsion than men. Their wills are not so strong. Many funny things have been said of "woman's will," but the strength of their will lies in its very weakness. It is a fact worthy of notice, that rotundity of outline, and symmetry of figure are the children of the spinal system; while activity and strength of the cerebral functions pare down the rotundity, and shapen the outline of the figure. Witness the shriveled limbs and sharpened faces of big-headed and precocious children. It is uncommon to find great beauty of person and strength of intellect in the same individual. Ever since Narcissus bending beside the fountain, became enamored of himself and changed into a daffodil, great beauties and great fools have been synonymous.

Female *savans* may have fine speaking countenances — faces which we *admire*, as we must admire intellect wherever found, but they are lacking in that softly moulded contour, that luxuriant but not redundant grace and fullness of figure, which we *love* in women and children. "Strong-minded women," with high cheek bones, "probulgent" foreheads, door-handle clavicles, and very scanty mammæ — the Mrs. Jellyby's of social life, whose "eyes are turned to Africa," or to the "missionary fields," or anywhere else save to their homes and firesides — with outline as angular and unyielding as a granite rock, are very little liable to convulsions. Through their compact forms the blood flows in scanty current through unyielding veins — the menstrual secretion is performed *guttatim* — congestive action is impossible.

There is another class of women, full to bursting with rich fibrinous blood, with bold dark eyes and hair, and a redundancy of figure which denotes strong sexual passions, who are peculiarly liable to convulsion. Their minds are generally of an inferior order, and so great is the predominance of the spinal system over the will and intellectual forces, that they are daily liable to the congestive and consequent convulsive action. Wet feet, or active exercise, or a little mental excitement are sufficient to break down the barriers of mental control, and subject the system to the unchecked influence of a spinal cord in full revolt from the government of the brain. But I have exceeded my limits. This vein of thought is but just opened — no divining rod is needed to see how great its influence might be, not only in our practice but in the great interests of education.

